# Prediction Equations Overestimate the Energy Requirements More for Obesity-Susceptible Individuals

**DOI:** 10.3390/nu9091012

**Published:** 2017-09-13

**Authors:** Rebecca T. McLay-Cooke, Andrew R. Gray, Lynnette M. Jones, Rachael W. Taylor, Paula M. L. Skidmore, Rachel C. Brown

**Affiliations:** 1 Department of Human Nutrition, University of Otago, P.O. Box 56, Dunedin 9054, New Zealand; rebecca.cooke@otago.ac.nz (R.T.M.-C.); paula.skidmore@otago.ac.nz (P.M.L.S.); 2 Nutrition Society of New Zealand, Whanganui 4543, New Zealand; 3 Department of Preventive and Social Medicine, University of Otago, P.O. Box 56, Dunedin 9054, New Zealand; andrew.gray@otago.ac.nz; 4 School of Physical Education, Sport and Exercise Sciences, University of Otago, P.O. Box 56, Dunedin 9054, New Zealand; lynnette.jones@otago.ac.nz; 5 Edgar Diabetes and Obesity Research Centre and Department of Medicine, Dunedin School of Medicine, University of Otago, P.O. Box 56, Dunedin 9054, New Zealand; rachael.taylor@otago.ac.nz

**Keywords:** obesity resistance, obesity susceptibility, resting metabolic rate, RMR prediction equations, indirect calorimetry

## Abstract

Predictive equations to estimate resting metabolic rate (RMR) are often used in dietary counseling and by online apps to set energy intake goals for weight loss. It is critical to know whether such equations are appropriate for those susceptible to obesity. We measured RMR by indirect calorimetry after an overnight fast in 26 obesity susceptible (OSI) and 30 obesity resistant (ORI) individuals, identified using a simple 6-item screening tool. Predicted RMR was calculated using the FAO/WHO/UNU (Food and Agricultural Organisation/World Health Organisation/United Nations University), Oxford and Miflin-St Jeor equations. Absolute measured RMR did not differ significantly between OSI versus ORI (6339 vs. 5893 kJ·d^−1^, *p* = 0.313). All three prediction equations over-estimated RMR for both OSI and ORI when measured RMR was ≤5000 kJ·d^−1^. For measured RMR ≤7000 kJ·d^−1^ there was statistically significant evidence that the equations overestimate RMR to a greater extent for those classified as obesity susceptible with biases ranging between around 10% to nearly 30% depending on the equation. The use of prediction equations may overestimate RMR and energy requirements particularly in those who self-identify as being susceptible to obesity, which has implications for effective weight management.

## 1. Introduction

Why some individuals remain lean with relative ease (ORI) while others continuously struggle with their body weight, despite living in a similar environment (OSI), is an intriguing question. However, little research has focused on comparing such groups, despite belief that this might yield novel information valuable for developing potential strategies to aid those who continually struggle with their weight. Resting metabolic rate (RMR) or basal metabolic rate (BMR) represents the largest component (60–75%) of total daily energy expenditure in sedentary humans [1]. Variations or modifications of RMR have the potential to influence energy balance and conceivably the susceptibility to gaining or maintaining body weight.

Observations from cross-sectional studies tend to show obese individuals have a higher RMR compared to healthy controls because of increased metabolically active mass [2,3,4,5,6,7]. However, once adjusted for variation in fat-free mass (FFM), most studies report similar RMRs between lean and obese subjects [5]. For individuals with a high body mass index (BMI), both FFM and fat mass make significant contributions to total body mass, so for this group, total body mass has been shown to be better correlated with RMR than FFM alone [8,9]. However, subsequent weight gain is generally associated with a low RMR at baseline [10,11]. Also, evidence suggests RMR is suppressed in conjunction with weight loss, often to a greater degree than would be expected based on changes in body weight/composition [12,13,14]. Known as adaptive thermogenesis or metabolic adaptation, this adaptive response reduces energy expenditure to effectively oppose the maintenance of reduced body weight [15,16] and is likely to contribute to the high rate of weight regain in overweight/obese persons after weight loss [14]. Therefore, it appears the relationship between obesity and RMR is dynamic, responding to changes in body weight and is dependent on when and how data are presented [10,17].

Direct and indirect calorimeters are the standard tools for assessing RMR in research settings. However, due to the expense of calorimeters, the time needed to achieve an accurate measurement, and the need for trained personnel to conduct the tests, the measurement of RMR for individual patients/clients is most commonly calculated from prediction equations [18,19,20]. Instead, the use of predictive equations developed through regression analyses, using calorimetry as the criterion measure and various characteristics of the individuals such as body mass, height, sex and age [18,19,21], is standard in dietetic practice [22,23]. There are many, varied equations available—a recent review identified 248 RMR estimation equations [24].

While some previous research has found prediction equations underestimate RMR [25], there is also evidence that prediction equations overestimate RMR [18,26,27]. Furthermore, there is debate as to the usefulness of many prediction equations [18]. In addition, a review which examined studies conducted between 1980 and 2000, suggested that in the majority of cases the FAO/WHO/UNU (Food and Agricultural Organisation/World Health Organisation/United Nations University) prediction equations overestimated RMR in a number of populations [28]. Also, Daly et al. [29] reported that energy requirements were overestimated using a number of different widely used prediction equations, including Harris-Benedict.

Due to the way they are generated, predictive regression equations work best in groups of people [18]. When these equations are applied to an individual (e.g., use of RMR equations to predict energy requirements in clinical dietetic practice), errors large enough to impact outcome could be produced especially if the individual does not share important characteristics with the group from whom the equation was developed (e.g., age, sex, body composition, ethnicity) [18]. For this reason, considerable debate surrounds the best equation for predicting metabolic rate in any particular setting, particularly in overweight or obese individuals [18,19,20,21,24,30]. In addition, whether these equations show any bias among those who are susceptible vs. those resistant to obesity is unknown, but important, given such knowledge is required to determine individual energy requirements for weight reduction. Only one study appears to have examined potential differences in RMR between these groups, reporting a lower absolute RMR in lean obesity resistant compared to obesity susceptible males (16.2% difference, *p* < 0.001). This difference disappeared when RMR was adjusted by fat mass and FFM (5.59 ± 0.97 vs. 5.61 ± 0.13 kJ·min^−1^) [31]. Whether RMR differs between those who are susceptible and resistant to obesity is becoming increasingly important because many more people are now exposed to RMR/BMR prediction equations due to the increasing popularity and use of diet tracking apps, such as “MyFitnessPal™”, that utilise these equations to generate energy intake targets. If these equations systematically over- or under-predict energy requirement goals there are potentially numerous clients, patients and app-users likely to experience disappointment and anxiety as they fail to meet the set healthy body weight targets.

The aim of this study was to compare absolute measurement of RMR in individuals specifically identified as obesity resistant or obesity susceptible. In particular, we aimed to compare the ability of three commonly used equations to predict RMR in these two distinct groups in order to identify whether two people of the same sex, age, weight, and height but each from one of these obesity resistant and susceptible groups might be subject to different biases compared to measured RMR.

## 2. Materials and Methods

### 2.1. Study Participants

Sixty-two participants were recruited from the general public in Dunedin, New Zealand, via advertisements placed in local newspapers, at supermarkets and in flyers that contained questions designed to specifically target obesity susceptible (OSI) and obesity resistant individuals (ORI). Eligible participants were healthy males or females, aged between 20 and 45 years meeting our criteria as either an OSI (struggles to maintain their weight, despite perceived low energy intakes) or as an ORI (remains lean with relative ease and can eat whatever they like) as previously defined [32,33]. Participants were classified as OSI if they answered positively to any of the statements outlined for OSI. Conversely, participants were classified as ORI if they answered positively to any of the statements outlined for ORI (Table 1).

Participants were excluded if they did not answer positively to any of the screening tool statements or if they answered positively to both an OSI and ORI statement, were smokers, pregnant, lactating, or menopausal, were currently taking stimulants or anti-depressants, had a previous history of an eating disorder, presence of a thyroid disorder or other medical condition/s that affect metabolic rate, or a thyroid stimulating hormone level outside the reference range (0.3–5 μIU·mL^−1^). Of the 76 respondents assessed for eligibility, 11 were excluded from entering the study. Ten were unable to be clearly classified as ORI or OSI and 1 had a previous history of an eating disorder. Thus 13% were neither ORI nor OSI. Two participants were retrospectively excluded from analysis, one due to diagnosis of a genetic condition that would influence study outcomes, and one due to a diagnosis of type 2 diabetes. A further person did not have their RMR measured. Obesity susceptible individuals (OSI) found it difficult to lose but not gain weight, were likely to experience fluctuations in weight (as indicated by self-reported weight history) and had a BMI of 21.6–44.0 kg·m^−2^. In contrast, ORI had always been lean (as indicated by self-reported weight history), found it difficult to gain but not lose weight and had a BMI of 17.5–27.7 kg·m^−2^.

The study was conducted in accordance with the Declaration of Helsinki, and the protocol was approved by the Human Ethics Committee of the University of Otago, New Zealand (08/005). All participants provided written informed consent prior to participation.

### 2.2. Body Composition

Body weight was measured in the fasting state in light clothing on calibrated electronic scales (Wedderburn, Tokyo, Japan) that measured to the nearest 0.1 kg. Height was measured to the nearest millimetre using a stadiometer with the head positioned in the Frankfort plane. Waist circumference (WC) was measured at the level of the narrowest point between the bottom of the 10th rib and the border of the iliac crest [34]. Body composition including fat mass, FFM and percentage body fat (%BF) was measured using dual-energy X-ray absorptiometry (DXA) (DPX-L Scanner, Lunar Corp., Cincinnati, OH, USA) using software version 1.35 (Lunar, Cincinnati, OH, USA) by a single experienced technician at the Dunedin Public Hospital DXA Scanning Unit.

### 2.3. Resting Metabolic Rate

Resting metabolic rate (RMR) was measured using indirect calorimetry based on best practice methods [35]. The assessment was conducted in an exercise physiology laboratory at the School of Physical Education, Sport and Exercise Sciences, University of Otago. All testing was conducted in a quiet, mildly lit and heated (average 20.5 °C) room. Environmental settings were kept consistent for all participants to ensure RMR measurements were not influenced by sound, light or temperature. Participants were asked to fast overnight and to abstain from alcohol and caffeine for at least ten hours and to avoid engaging in strenuous activity for 24 h prior to the test. Participants were instructed to take any daily prescribed medication as usual. Testing was undertaken between 6:00 a.m. and 10:00 a.m. to ensure participants were not fasting for long periods of time during waking hours. Menstruating female participants were measured during the follicular phase of the menstrual cycle to avoid the thermic effect of progesterone on metabolic rate during the luteal phase [36,37,38].

Participants rested for 15 min in a semi-recumbent position and were instructed not to fall asleep. Participants then breathed through a face-mask for 15 min during which time expired gases were collected using a Sensormedics 2900 metabolic cart (Sensormedics, Yorba Linda, CA, USA). Gas analysers were calibrated prior to each test using set O_2_ (26%) and O_2_/CO_2_ (16% and 4%) mixtures before testing began each morning and again if more than two tests were carried out in one morning. Volume was calibrated before each test using a standard 3 L syringe. Breakfast was provided to participants following testing.

The first five min of data collection were discarded and the remaining 10 min used to determine a 4 min period having a coefficient of variation (CV) for $\dot{V}O_{2}$ (L·min^−1^) and $\dot{V}{\mathrm{CO}}_{2}$ (L·min^−1^) of ≤ 10% for analysis. If these criteria were not reached (*n* = 22), values for the lowest CV were used in the analysis. Four female participants (3 ORI, 1 OSI) and one male participant (OSI) had a respiratory exchange ratio (RER) value above 1, indicating pre-test protocol violations or measurement inaccuracy and so were excluded from the analysis. The abbreviated Weir equation [39] was used to determine RMR from mean $\dot{V}O_{2}$ (L·min^−1^) and $\dot{V}{\mathrm{CO}}_{2}$ (L·min^−1^) as used previously [40]:

$$\mathrm{RMR}\;(\mathrm{kcal}\cdot d^{-1})\;=\;(3.941\;(\dot{V}O_{2})\;+\;1.106\;(\dot{V}{\mathrm{CO}}_{2}))\;\times \;1440.$$

### 2.4. Predictive Equations for Estimating Resting Metabolic Rate (RMR)

Predicted RMR was calculated using 3 well known previously published prediction equations: the FAO/WHO/UNU (Food and Agricultural Organisation/World Health Organisation/United Nations University) equation [41], the Oxford equation [28] and the Mifflin-St Jeor equation [42] and compared to measured RMR. These particular equations were selected for study, because of their popular use by health professionals (e.g., Dietitians New Zealand [22] and the Academy of Nutrition and Dietetics [23]) and their use in diet tracking apps to set energy intake targets.

### 2.5. Physical Activity

Participants were instructed to wear an Actical accelerometer (Mini Mitter Co Inc., Bend, OR, USA) on the right hip at waist level for at least seven consecutive days at all times except when sleeping, or engaging in activities potentially harmful to the device (e.g., contact or combat sports). Accelerometer data were scored and interpreted using the MeterPlus Version 4.3 software from Santech, Inc. (San Diego, CA, USA) [43]. Quality control and data reduction procedures used in the analysis of accelerometer data from the National Health and Nutrition Examination Survey (NHANES) [44] and the Canadian Health Measures Survey (CHMS) [45] were applied. A spurious data threshold of 20,000 counts per minute (cpm) was used. A valid day was defined as having ≥ 10 h of accelerometer wear time while non-wear time was defined by a period of at least 60 consecutive minutes of zero activity counts, with allowance for 1 to 2 min of counts between 0 and 100. Participants with ≥4 valid days of data were retained for analysis. Mean cpm and time spent in various levels of movement intensity using sedentary [46], light, moderate and vigorous [47] cut-points specific to the Actical accelerometer, were determined.

### 2.6. Dietary Assessment

Participants received detailed verbal and written instructions from a trained researcher on how to collect a weighed four-day diet record (4DDR). All food and beverage intakes were recorded at the time of consumption on non-consecutive days (3 week days, 1 weekend day). The completed 4DDRs were analysed using “Kai-culator” (version 1.08, Department of Human Nutrition, University of Otago, Dunedin, Otago, New Zealand) the dietary assessment software developed in the Department of Human Nutrition, University of Otago. The food composition database includes current and previous versions of FOODfiles (2010v2) from Plant and Food Research Ltd and selected recipes calculated from the 2008/09 New Zealand Adult Nutrition Survey [48]. All diet record information was entered by one well-trained researcher and comprehensive and detailed notes regarding food and beverage item substitutions were maintained throughout.

A sensitivity analysis was undertaken to examine the impact of low energy reporting. Participants with an energy intake to RMR ratio (EI:RMR) of <1.06 were classified as low energy reporters (LER) using the Goldberg method as outlined by Gibson [49].

### 2.7. Statistical Analysis

Appropriate summary statistics were calculated for both the obesity resistant and susceptible groups. These variables were compared between the susceptibility groups using two-sample *t*-tests where the model assumptions were satisfied for continuous variables (approximate normality and homogeneity of residuals) and Mann-Whitney U tests otherwise for continuous variables, and Chi-squared (where at least 80% of cells had expected cell counts of 5 or greater) or Fisher’s Exact (otherwise) tests for categorical variables. Ratios of each estimated RMR compared to RMR derived from indirect calorimetry were calculated and modelled for each estimation equation interacting with obesity susceptibility (i.e., six combinations of equation and susceptibility) using linear mixed models, with a random subject effect to accommodate the clustering within individuals who each provided all three ratios. These models also included estimated RMR as a covariate, which was allowed to interact with the six pairs described above (i.e., as a three-way interaction between RMR, equation, and susceptibility). To incorporate potential non-linearities with the continuous derived RMR measure, the addition of a quadratic term was investigated and retained (based on improvements in Akaike Information Criterion AIC), as was the subsequent addition of a cubic term. All 56 participants were used for each model. Due to the sample size, interactions between quadratic and cubic terms and the estimation equation and susceptibility were not investigated. Log-transformations were used for the ratios (as would be anticipated) and this improved model residuals. Restricted maximum likelihood was used for all models. In order to determine whether any differences in these ratios could reflect biases in the equations by susceptibility categories, the equation parameters (age, sex, height, and weight) were then added to the model as main effects only (in particular, not allowing age, height, and weight effects to vary by sex which is the case in the RMR equations). All statistical analyses were performed using Stata 14.2 (StataCorp LLC, College Station, TX, USA) and two-sided *p* < 0.05 was considered statistically significant in all cases.

## 3. Results

### 3.1. Participant Characteristics

The characteristics of the study participants are shown in Table 2.

As expected, OSI were significantly heavier, had a higher body mass index (BMI), fat mass and %BF, and a larger WC compared to ORI. The percentage of males and females, age, height and FFM was similar between OSI and ORI. There were no significant differences in the amount of time participants spent participating in various levels of movement intensity or engaging in sedentary behaviours.

### 3.2. Dietary Intake

No significant differences were observed for absolute energy intake or any of the energy-yielding nutrients expressed as a percentage of total energy intake. When energy intake was expressed relative to body weight, OSI had a significantly lower intake than ORI (*p* < 0.001). The results of the sensitivity analysis revealed that two participants were classified as low energy reporters (LER), defined as EI:RMR <1.06. One LER was an obesity susceptible female and one was an obesity resistant female. Removal of the two LER from the analysis of the dietary intake data did not affect the results and data for the full sample has been reported.

### 3.3. Weight History

Those in the OSI group were more likely to have changed their eating habits in an attempt to lose weight (69% versus 13%, Chi-square *p* < 0.001). Comparing the number of attempts between the four ORI and 18 OSI participants reporting such attempts, with the number of attempts ranging between 1 and 13, did not find evidence of differences between these groups (Mann-Whitney U *p* = 0.223). Those in the ORI group were more likely to report having changed their eating habits to gain weight (47% versus 0%, Fisher’s Exact *p* < 0.001) with a maximum of two attempts reported. Those in the susceptible group had higher lightest (*p* = 0.020) and heaviest (*p* < 0.001) body weights and appreciably greater weight ranges (a median of 25.0 kg vs. 8.0 kg, *p* < 0.001) compared to ORI.

### 3.4. Resting Metabolic Rate (RMR)

No significant differences in absolute RMR or RMR expressed relative to FFM were observed amongst OSI and ORI, and there was substantial overlap between the two groups in terms of the distribution of RMR.

**Figure 1:**
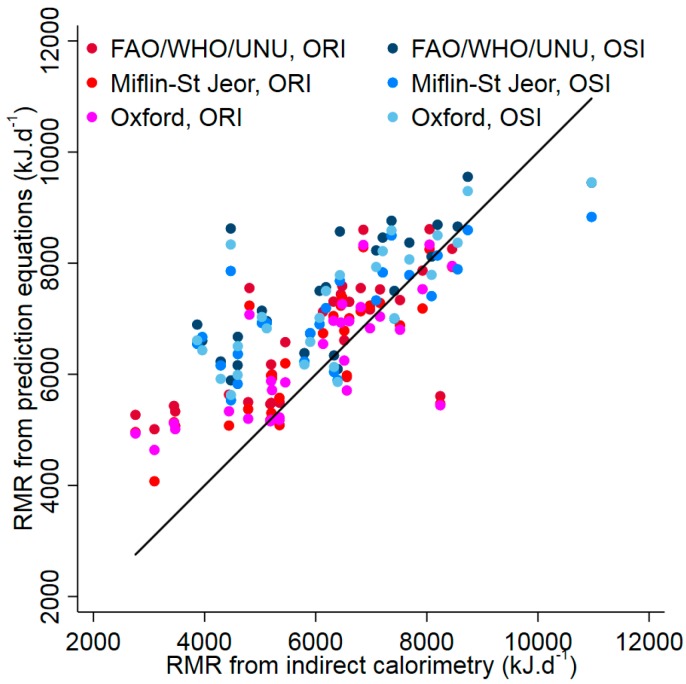
Scatter plot of predicted against measured RMR for each equation for obesity resistant individuals (ORI) and obesity susceptible individuals (OSI). Each individual is represented by three points and the line shows equality between the prediction equation and measured RMR values.

**Figure 2:**
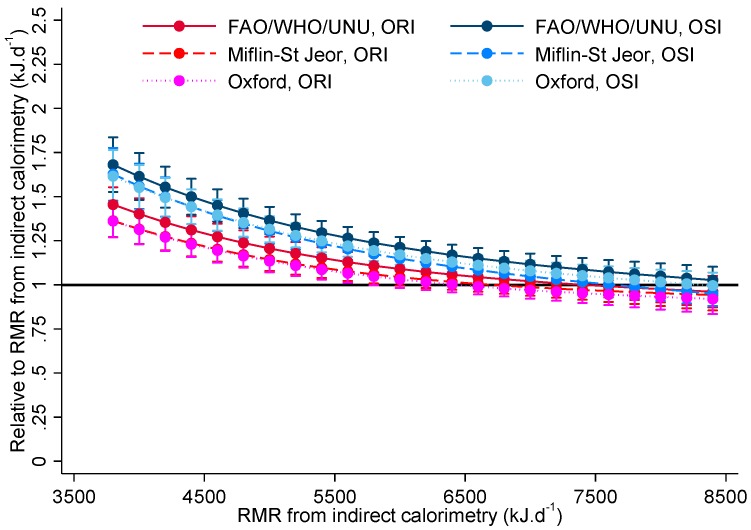
Biases for obesity resistant individuals (ORI) and obesity susceptible individuals (OSI) by resting metabolic rate (RMR) prediction equation for selected measured RMR values (*n* = 56).

Figure 1 shows a scatterplot of predicted RMR against measured RMR which also illustrates the high degree of overlap in measured RMR between ORI and OSI. As can be seen in Figure 2, with the association between measured RMR and the bias including a cubic term, all three equations overestimated RMR for both obesity susceptible and resistant individuals when measured RMR was 5000 kJ·d^−1^ or less. A suggestion of underestimation can be seen when measured RMR is 11,000 kJ·d^−1^ or greater, although this was not consistently statistically significant.

Table 3 shows the relative bias for OSI versus ORI by equation for selected measured RMR values. Across all three equations, almost all results for measured RMRs 7000 kJ·d^−1^ or lower show statistically significant evidence that the equations overestimate RMR to a greater extent for those classified as susceptible with biases ranging between around 10% to nearly 30% depending on equation and measured RMR. Similar patterns of bias were found when using RMR normalised by fat free mass, where those with lower values had their RMRs overestimated by all three equations and with a greater bias for those in the susceptible group, although the magnitude of the bias was reduced and some biases for lower values were tendencies (0.05 < *p* < 0.10) rather than statistically significant for the WHO equation.

**Figure 3:**
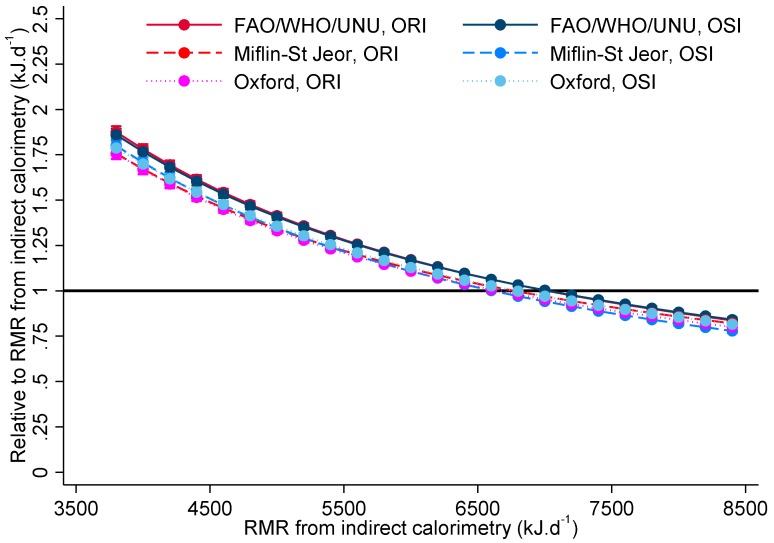
Biases for obesity resistant individuals (ORI) and obesity susceptible individuals (OSI) by resting metabolic rate (RMR) prediction equation for selected measured RMR values after adjustment for sex, age, height and weight (*n* = 56).

After further adjusting for sex, age, height, and weight (Figure 3), the biases overall remain evident for lower measured RMRs (6000 kJ·d^−1^ and below) with an under-estimation bias evident for measured RMRs 8000 kJ·d^−1^ and above. However, the differences between methods and between susceptibility groups by method are no longer evident. Adding physical activity as total hours per day of moderate or vigorous activity did not meaningfully alter these results.

As shown in Table 4, there was no evidence of a different bias by susceptibility for the FAO/WHO/UNU equation at any level of measured RMR and biases for the Oxford equation were greatly attenuated (2% or less) and only significant between 5000 kJ·d^−1^ and 7000 kJ·d^−1^. The biases from the Miflin-St Jeor equation continued to differ by susceptibility, although these were again attenuated from the unadjusted analyses, displaying a pattern of greater overestimation for those susceptible where measured RMR was 3000 kJ·d^−1^ and below and under-estimation when derived RMR was 7000 kJ·d^−1^ and above.

## 4. Discussion

In the present study, indirect calorimetry was used to measure the RMR of individuals who struggle to maintain a healthy body weight (OSI) compared to individuals who maintain their body weight with relative ease (ORI). In contrast to findings from the majority of previous cross-sectional studies where absolute RMR was shown to be greater in obese versus non-obese controls [2,3,4,5,6,7], no significant differences were observed in absolute RMR amongst OSI compared to ORI. A likely explanation for this disparity in results relates to how the participants were defined in the current study. Rather than being classified based solely on BMI or body weight, participants were identified based on their resistance or susceptibility to obesity in terms of the ease or difficultly they have maintaining a healthy body weight. As a consequence the body weight and BMI difference between the two groups, although statistically significant, is likely to be less than when participants are classified as obese and non-obese. A large difference in body weight/BMI is likely to contribute to a difference in absolute RMR.

Previous research found lower RMRs in lean versus obese participants [5]. In our study, there was no evidence of a difference in RMR between the related groups of ORI and OSI. Recent evidence shows that organs such as the brain, heart, liver and kidneys as well as fat and skeletal muscle mass contribute significantly to resting energy expenditure [50]. Obesity susceptible individuals (OSI) had a significantly greater quantity of fat mass contributing to total body weight compared to ORI. Therefore, the metabolic activity of a given unit of body weight would be expected to be lower in this group compared to ORI. This disparity in RMR could lead to substantial increases in body weight over time, if compensatory behaviours such as reducing energy intake or increasing energy expenditure were not initiated. Similarly, the authors of [31] found evidence of differences but not after normalising RMR to body weight or fat free mass. Predicted weight gain is estimated to be around 7.4 kg over a 12 month period (average for this group) according to the Pennington Biomedical Research Centre calculator [51], which uses a dynamic human weight change prediction model developed by Thomas and colleagues [52]. Therefore, a low relative RMR for a given body size or composition may increase the risk of sustained positive energy balance and subsequent weight gain [12,53].

In the present study, OSI indicated the need to consume smaller amounts to manage their weight (100% of OSI females; 64% of OSI males) (Table 1) based on their response to the screening tool statements. This seems to be corroborated by a lower RMR relative to body weight compared to ORI. Taken together, the lower relative energy intake combined with evidence of a lower relative RMR could represent confirmation of this subjective perception in OSI. Underreporting of energy intake is a common source of measurement error in dietary assessment [49,54,55]. Low energy reporting was assessed in the present study using measured RMR and applying appropriate Goldberg cut-off values [49]. In the present study only two of the 56 participants included in the dietary analysis were classified as LER by having an (EI:RMR) of <1.06. Removal of these two participants from the analysis did not affect the group energy intake results. It is therefore unlikely that low energy reporting is responsible for the lower relative energy intake of OSI compared to ORI in the present study.

By definition, OSI (individuals who self-identify they struggle to maintain a healthy body weight) are likely to have gained or lost weight in the past and this may be one explanation for the differences in RMR between the two groups in the present study. Previous research suggests RMR is suppressed in conjunction with weight loss, often to a greater extent than would be expected based on changes in body weight/body composition [12,13,14]. Arguably the most successful dieters on the planet, competitors in the “The Biggest Loser™” television programme with the greatest weight loss at the end of the competition also experienced the greatest slowing of RMR [14]. Six years following the end of the show, those who were most successful at maintaining weight loss experienced the greatest metabolic slowing, despite continuing to engage in high levels of exercise [13]. In addition, metabolic suppression persisted even in those who experienced substantial weight regain in the intervening 6 year period [13]. Therefore, due to past fluctuations in body weight amongst the OSI, which were three times greater than for ORI individuals, these individuals in the present study may be exhibiting metabolic adaptation—an adaptive response that reduces energy expenditure to oppose the maintenance of a reduced body weight [15,16].

Resting metabolic rate is an important issue to consider within the area of weight control, as it is this component that accounts for the greatest percentage of total daily energy expenditure. Clinical nutrition management frequently relies on predicting RMR from equations that use various combinations of anthropometric, age and gender measures. In the present study, important differences were observed between measured and predicted RMR from the selected prediction equations. All three equations overestimated RMR for both OSI and ORI when measured RMR was ≤5000 kJ·d^−1^. In addition, across all three equations, there was evidence that when measured RMRs are ≤7000 kJ·d^−1^, the equations overestimate RMR to a greater extent for those classified as susceptible with biases ranging between around 10% to nearly 30%. We found that adjusting the model of predicted RMR for the components of the equations (weight, height, age, and sex) greatly attenuated the biases between OSI and ORI, suggesting that further calibration of the equations might effectively remove the clinically significant biases between OSI and ORI, although we have no evidence that this would remove the overall biases of the equations compared to RMR derived from indirect calorimetry. Another possible interpretation is that our observed differences in RMR misestimation between self-reported obesity susceptible and resistant phenotypes can be largely explained by these variables alone without reference to additional mechanisms.

These findings have important implications for dietetic practice. The Oxford equation has recently been adopted by Dietitians New Zealand and is recommended for calculating RMR in the latest edition of the Clinical Handbook [22]. Meanwhile, the Academy of Nutrition and Dietetics (formally the American Dietetic Association) recommends using the Miflin-St Jeor equation for estimating RMR in overweight and obese individuals [23]. As these equations predominantly over estimate RMR to a greater extent in this group who struggle with maintaining a healthy body weight, their use in dietary counseling is likely to lead to an over-prediction of total energy requirements. Therefore, calculation of energy restriction based on these over predicted energy requirements may be insufficient to facilitate meaningful weight maintenance/loss in this group, leading to disappointment and anxiety as clients/patients fail to meet their healthy body weight targets. In addition, the Miflin-St Jeor equation is used to estimate RMR and from there to predict energy requirements as part of the self-monitoring dietary intake apps “MyFitnessPal™” [56], “Nutrino™” [57] and Fitday™ [58]. With millions of users reported worldwide (“MyFitnessPal™” ≥80 million; “Nutrino™” >6 million) [59,60] including, undoubtedly, a fair proportion who could be defined as OSI, there are potentially numerous users who may be wondering why they are unable to achieve the weight loss targets predicted by these apps.

To provide some perspective on the magnitude of over-estimation of RMR for OSI and ORI, and subsequent energy imbalance using these popular equations, we present a variety of scenarios. For a person with an RMR of 5000 (measured by indirect calorimetry), the Miflin-St Jeor equation would overestimate RMR by 14% (on average) for an ORI and 31% (on average) for an OSI, equivalent to 717 kJ·d^−1^ and 1531 kJ·d^−1^ respectively. According to the Pennington Biomedical Research Centre calculator [51] an energy intake based on this overestimation, would lead to weight gains of 3.7 (ORI) and 7.6 (OSI) kg over 12 months for a 33 year old female who was 69 kg and 1.66 m (typical values for a female participant with an estimated RMR 5000 ± 5000 kJ·d^−1^). The additional weight gain of the OSI would be 3.9 kg compared to the equivalent ORI. Similarly, for an RMR of 6000 kJ·d^−1^, the Miflin-St Jeor equation would overestimate by 4% (ORI) and 15% (OSI), equivalent to 262 kJ·d^−1^ and 896 kJ·d^−1^, respectively. For a 33 year old male who was 75 kg and 1.78 m, this would lead to weight gains of 1.5 (ORI) and 4.2 (OSI) kg over a year, an additional 2.7 kg gain for the OSI. Similar results apply to the other two equations.

Given that these equations are overestimating energy requirements, especially among OSI, future research using large samples should focus on developing equations specific to OSI and/or calibrating existing equations, possibly by adding additional components such as percentage body fat. If successful, such approaches would be invaluable for healthcare practitioners and researchers working in this area.

An important strength of this study include the use methods considered to be the gold standard for each variable, including DXA to assess body composition, weighed food records to assess dietary intake and accelerometry to provide an objective measure of physical activity. However, some limitations need to be considered when interpreting the results of the current study. Firstly, individual-level matching by age, sex, physical activity or other lifestyle factors for OSI and ORI was not possible. Resting metabolic rate is affected by age and decreases 1–2% per decade after 20 years of age [61]. However, OSI and ORI participants were overall similar in terms of age, sex and height and differences in their physical activity were minimal. Furthermore, age and sex are included in the RMR prediction equations. The RMR of individuals who exercise regularly is generally found to be higher than non-exercisers [62] and in particular resistance training has an impact on RMR due to its role in increasing FFM [62,63]. Although physical activity levels were similar between OSI and ORI in the present study, the methods used to assess physical activity did not provide specific information on engagement in resistance exercise and it is therefore unknown to what degree differences in resistance exercise participation may have had on RMR. Also, while the screening tool was developed based on an extensive review of the literature and appears to have face and content validity, and demonstrated some concurrent validity with regards to current weight and weight history, it has not been formally validated and it is plausible that it did not adequately differentiate ORI and OSI.

## 5. Conclusions

In conclusion, commonly used prediction equations overestimated the RMR of both OSI and ORI, but more so in OSI. This is of concern for OSI using self-monitoring dietary-intake apps for weight loss or following planned energy-restriction programmes prescribed during nutrition counseling, as the weight loss targets may be impossible to achieve using the energy requirements over-estimated by these prediction equations. It is difficult to overcome this issue for individuals using self-monitoring dietary-intake apps, but identification of OSI prior to implementation of planned energy restriction in a nutrition counseling setting could allow a potentially lower energy requirement to be factored in for these clients/patients. Future investigations may explore the development of RMR prediction equations that include calibration for obesity susceptibility. Such equations would be valuable in providing appropriate energy intake targets for weight loss for OSI.

## Figures and Tables

**Table 1:** Screening statements for classification of participants as obesity susceptible (OSI) or obesity resistant individuals (ORI).

Statements for OSI	Statements for ORI
1. I am a person who needs to eat small amounts of food to manage my weight	1. I am a person who can eat whatever I like without gaining weight
2. I am a person who gains weight easily	2. I am a person who loses weight easily
	3. I am a person who maintains my weight easily
	4. I am a person who finds it difficult to put on weight

**Table 2:** Characteristics of obesity susceptible individuals (OSI) and obesity resistant individuals (ORI).

Variable	OSI (*n* = 26)	ORI (*n* = 30)	*p*-Value
Sex			
Female	14 (54%)	14 (47%)	
Male	12 (46%)	16 (53%)	0.592 ^a^
Age *	35.6 (8.1)	32.4 (7.8)	0.135 ^b^
Anthropometrics			
Height (m) *	1.70 (0.10)	1.70 (0.10)	0.322 ^b^
Weight (kg) *	89.5 (14.0)	66.0 (12.4)	<0.001 ^b^
BMI (kg/m^2^) ^¶^	29.9 (26.5, 33.2)	21.5 (19.7, 23.2)	<0.001 ^c^
WC (cm) ^¶^	98.2 (87.3, 106.3)	77.8 (71.8, 81.8)	<0.001 ^c^
Body Composition			
Fat Mass (kg) ^¶^	30.7 (23.9, 38.3)	12.7 (9.0, 16.0)	<0.001 ^c^
FFM (kg) ^¶^	51.9 (45.0, 62.2)	47.8 (38.4, 58.1)	0.153 ^c^
Percentage Body Fat (%) ^¶^	35.2 (27.2, 43.9)	21.8 ((13.8, 24.6)	<0.001 ^c^
Physical Activity			
Sedentary (h·d^−1^) ^¶^	10.7 (9.8, 11.1)	11.2 (9.9, 12.0)	0.238 ^c^
Light (h·d^−1^) ^¶^	3.5 (3.0, 3.7)	3.0 (2.5, 3.7)	0.207 ^c^
Moderate (h·d^−1^) ^¶^	0.5 (0.4, 0.8)	0.6 (0.4, 0.9)	0.341 ^c^
Vigorous (h·d^−1^) ^¶^	0.0 (0.0, 0.2)	0.2 (0.0, 0.4)	0.070 ^c^
Dietary Intake			
Energy (kJ·d^−1^) ^¶^	9803 (8379, 12,203)	11467 (9581, 12,913)	0.119 ^c^
Energy (kJ·kgBW^−1^·d^−1^) ^¶^	121 (100, 132)	172 (149, 196)	<0.001 ^c^
Protein (%TEI) ^¶^	17.6 (15.3, 19.3)	15.6 (13.7, 17.8)	0.152 ^c^
Fat (%TEI) ^¶^	32.3 (27.8, 35.4)	34.2 (29.9, 37.1)	0.359 ^c^
CHO (%TEI) ^¶^	46.4 (42.2, 50.0)	47.6 (44.6, 50.2)	0.340 ^c^
SFA (%TEI) ^¶^	12.0 (10.4, 15.5)	12.7 (10.9, 15.7)	0.646 ^c^
MUFA (%TEI) ^¶^	11.5 (9.2, 12.9)	12.1 (10.4, 13.6)	0.313 ^c^
PUFA (%TEI) ^¶^	4.5 (3.7, 6.1)	4.7 (4.0, 5.7)	0.883 ^c^
Sugar (%TEI) ^¶^	21.3 (16.2, 25.7)	20.1 (18.7, 24.4)	0.985 ^c^
Alcohol (%TEI) ^¶^	0.1 (0.0, 3.9)	0.0 (0.0, 1.2)	0.138 ^c^
Eating Frequency			
Eating Occasions (*n*·d^−1^) ^¶^	4.4 (3.5, 4.9)	4.5 (3.9, 5.6)	0.156 ^c^
Weight History			
Weight loss attempts 0	8 (31%)	26 (87%)	<0.001 ^d^
1	3 (12%)	2 (7%)	
2–3	9 (35%)	1 (3%)	
4–9	4 (15%)	1 (3%)	
10+	2 (8%)	0 (0%)	
Weight gain attempts 0	26 (100%)	16 (53%)	<0.001 ^e^
1	0 (0%)	9 (30%)	
2	0 (0%)	5 (17%)	
Lightest weight (kg) * 67.7 (10.7)		59.9 (13.0)	0.020 ^b^
Heaviest weight (kg) * 96.9 (17.5)		70.1 (13.5)	<0.001 ^b^
Individual weight fluctuation (kg) ^¶^ 25.0 (14.0, 38.0)		8.0 (6.0, 13.0)	<0.001 ^c^
RMR (indirect calorimetry)			
Absolute (kJ·d^−1^) *	6339 (1752)	5893 (1520)	0.313 ^b^
RMR (prediction equations)			
FAO/WHO/UNU (kJ·d^−1^) *	7545 (1109)	6609 (1103)	0.003 ^b^
Miflin-St Jeor (kJ·d^−1^) *	7108 (906)	6334 (1110)	0.007 ^b^
Oxford (kJ·d^−1^) *	7291 (1100)	6253 (1080)	<0.001 ^b^

* Mean (SD), ^¶^ median (25th percentile, 75th percentile), ^a^ Chi-squared, ^b^ Two sample *t* test, ^c^ Mann-Whitney U, ^d^ Chi-squared test comparing none versus any, ^e^ Fisher’s exact test comparing none versus any. Abbreviations: % = percent, BMI = body mass index, BW = body weight, CHO = carbohydrate, FAO/WHO/UNU = Food and Agricultural Organisation/World Health Organisation/United Nations University, FFM = fat-free mass, h = hour, MUFA = monounsaturated fatty acids, *n* = number, ORI = obesity resistant individuals, OSI = obesity susceptible individuals, PUFA = polyunsaturated fatty acids, RMR = resting metabolic rate, SD = standard deviation, SFA = saturated fatty acids, TEI = total energy intake, WC = waist circumference.

**Table 3:** Relative bias for obesity resistant individuals (ORI) versus obesity susceptible individuals (OSI) by resting metabolic rate (RMR) prediction equation for selected measured RMR values.

Estimated RMR from Indirect Calorimetry (kJ·d^−1^)	Difference between OSI and ORI for FAO/WHO/UNU	*p*-Value	Difference between OSI and ORI for Oxford	*p*-Value	Difference between OSI and ORI for Miflin-St Jeor	*p*-Value
2000	1.19 (1.00, 1.42)	0.052	1.23 (1.03, 1.47)	0.021	1.27 (1.07, 1.52)	0.007
3000	1.17 (1.02, 1.34)	0.025	1.21 (1.05, 1.38)	0.008	1.23 (1.07, 1.41)	0.003
4000	1.15 (1.04, 1.28)	0.007	1.18 (1.07, 1.31)	0.001	1.18 (1.07, 1.31)	0.001
5000	1.13 (1.05, 1.22)	0.001	1.16 (1.08, 1.25)	<0.001	1.14 (1.06, 1.23)	<0.001
6000	1.11 (1.05, 1.18)	<0.001	1.14 (1.07, 1.20)	<0.001	1.10 (1.04, 1.17)	0.001
7000	1.09 (1.02, 1.17)	0.012	1.11 (1.04, 1.19)	0.003	1.06 (0.99, 1.14)	0.094
8000	1.08 (0.97, 1.19)	0.149	1.09 (0.99, 1.20)	0.084	1.02 (0.93, 1.13)	0.646
9000	1.06 (0.92, 1.21)	0.415	1.07 (0.94, 1.22)	0.325	0.99 (0.86, 1.13)	0.845
10,000	1.04 (0.88, 1.24)	0.658	1.05 (0.88, 1.25)	0.589	0.95 (0.80, 1.13)	0.569
11,000	1.02 (0.83, 1.26)	0.839	1.03 (0.83, 1.27)	0.798	0.92 (0.74, 1.13)	0.422

Differences are ratios of geometric means (95% CI): values greater than 1.00 indicate that RMR is overestimated in OSI compared with ORI, values lower than 1.00 indicate that RMR is underestimated in OSI compared with ORI. CI = confidence interval, FAO/WHO/UNU = Food and Agricultural Organisation/World Health Organisation/United Nations University, ORI = obesity resistant individuals, OSI = obesity susceptible individuals, RMR = resting metabolic rate.

**Table 4:** Relative bias for obesity resistant individuals (ORI) versus obesity susceptible individuals (OSI) by resting metabolic rate (RMR) prediction equation for selected measured RMR values after adjustment for sex, age, height and weight.

Estimated RMR from Indirect Calorimetry (kJ·d^−1^)	Difference between OSI and ORI for FAO/WHO/UNU	*p*-Value	Difference between OSI and ORI for Oxford	*p*-Value	Difference between OSI and ORI for Miflin-St Jeor	*p*-Value
2000	0.99 (0.95, 1.02)	0.489	1.02 (0.98, 1.06)	0.310	1.06 (1.02, 1.09)	0.004
3000	0.99 (0.96, 1.02)	0.493	1.02 (0.99, 1.05)	0.213	1.04 (1.01, 1.07)	0.013
4000	0.99 (0.97, 1.02)	0.512	1.02 (1.00, 1.04)	0.112	1.02 (1.00, 1.05)	0.068
5000	1.00 (0.98, 1.01)	0.576	1.02 (1.00, 1.04)	0.040	1.01 (0.99, 1.02)	0.569
6000	1.00 (0.98, 1.01)	0.757	1.02 (1.00, 1.03)	0.016	0.99 (0.97, 1.00)	0.139
7000	1.00 (0.98, 1.02)	0.968	1.02 (1.00, 1.03)	0.030	0.97 (0.96, 0.99)	0.001
8000	1.00 (0.98, 1.02)	0.778	1.02 (1.00, 1.04)	0.097	0.96 (0.94, 0.98)	<0.001
9000	1.01 (0.98, 1.03)	0.682	1.02 (0.99, 1.05)	0.208	0.94 (0.92, 0.97)	<0.001
10,000	1.01 (0.97, 1.04)	0.633	1.02 (0.98, 1.05)	0.325	0.93 (0.90, 0.96)	<0.001
11,000	1.01 (0.97, 1.05)	0.605	1.02 (0.98, 1.06)	0.425	0.91 (0.87, 0.95)	<0.001

Differences are ratios of geometric means (95% CI): values greater than 1.00 indicate that RMR is overestimated in OSI compared with ORI, values lower than 1.00 indicate that RMR is underestimated in OSI compared with ORI. CI = confidence interval, FAO/WHO/UNU = Food and Agricultural Organisation/World Health Organisation/United Nations University, ORI = obesity resistant individuals, OSI = obesity susceptible individuals, RMR = resting metabolic rate.

## Abbreviations

%	percent
BMI	body mass index
BW	body weight
CHO	carbohydrate
CI	confidence interval
d	day
FAO/WHO/UNU	Food and Agricultural Organisation/World Health Organisation/United Nations University
FFM	fat-free mass
h	hour
MUFA	monounsaturated fatty acids
*n*	number
ORI	obesity resistant individuals
OSI	obesity susceptible individuals
PUFA	polyunsaturated fatty acids
RMR	resting metabolic rate
SD	standard deviation
SFA	saturated fatty acids
TEI	total energy intake
WC	waist circumference.
